# The Impact of Labor Force Participation on Elderly Health in China

**DOI:** 10.3390/healthcare11020160

**Published:** 2023-01-04

**Authors:** Jianming Hou, Wenjian Zhou, Kefei Zhu, Xiaofei Ren

**Affiliations:** 1 Center for Northeast Asian Studies, Jilin University, Changchun 130012, China; 2 Northeast Asian Studies College, Jilin University, Changchun 130012, China; 3 Institute of Northeast Asian Studies, Heilongjiang Academy of Social Sciences, Harbin 150016, China

**Keywords:** labor force participation, health in old age, problem of endogeneity

## Abstract

In the context of the deepening of population aging and the trial implementation of a progressive retirement delay policy in China, understanding the relationship between the labor force participation and health status of the elderly will not only enrich relevant research but also help the elderly better achieve their goals of active aging and aging. Using the 2018 China Health and Retirement Longitudinal Study, this paper first established multiple linear regression models to analyze the impact of labor force participation on the health status of elderly people in China and then established simultaneous equation models using households living on minimum living allowances and the community average of labor participation as instrumental variables to deal with the endogeneity caused by two-way causality. The findings confirmed significant positive correlations between labor force participation and physical and mental health, while caring for grandchildren and participating in social activities were found to be negatively moderated the relationship between labor force participation and the physical and mental health of older adults. The impact of labor force participation on the physical health status of older men and the mental health status of older women may be greater. In addition, labor force participation may have a greater impact on the physical health of the rural elderly, and its impact on mental health was not found to be statistically significant between urban and rural areas.

## 1. Introduction

Due to the deepening of population aging in China, it has become to improve the health status of the elderly. The improvement of medical standards, changes in the concept of fertility, the extended healthy life expectancy of the elderly, and the declining number of births have led to increasing proportions of the elderly in China year by year. According to the sixth and seventh national census, the proportion of the population aged 60 and above in China increased from 13.26% in 2010 to 18.7% in 2020 and the proportion of the population aged 65 and above in China increased from 8.87% in 2010 to 13.50% in 2020 [1,2]. However, an increasing number of older people is experiencing physical and mental health problems as their physical functions continue to deteriorate. In order to achieve the goal of healthy aging and establish the concept of active aging, the Central Committee of the Communist Party of China and The State Council proposed in November 2021 to strengthen the Party’s overall leadership on aging work, adhere to the people-centered approach, and implement the national strategy of actively coping with population aging [3]. In December 2021, The State Council put forward specific requirements for promoting healthy aging [4]. In April 2022, The State Council vowed to accelerate the improvement of national health policies, continuously promote the construction of a healthy China, and constantly meet the growing health needs of the people [5].

Research on labor force participation is of great theoretical and practical significance to the health status of the elderly. In terms of theoretical significance, unlike previous studies, this paper not only focuse on the effect of labor force participation on older adults’ health status and provides new evidence for the divergence of views on its direction of influence but also considers the existence of two-way causality between the two and the endogeneity it entails. This study additionally examines the heterogeneity of this effect across groups and provides a new perspective for understanding the impact of labor force participation on older adults’ health. From a practical point of view, this study clarifies the influence of the labor force participation of the elderly on their health status, which could be of great help to the elderly in finding suitable social roles and satisfying their higher-level needs, and provides a reference for optimizing the national policy of delaying retirement and solving the labor shortage problem. Furthermore, this study could also help the elderly develop their labor resources and open the second demographic dividend, as well as provide a theoretical basis for the country to better cope with its aging problem.

The hierarchy of needs theory divides human needs Into five levels: physiological needs, safety needs, belongingness and love needs, esteem needs, and self-actualization needs [6]. Under normal circumstances, the elderly want to participate in society, gain social recognition and respect, and realize their own value after their basic needs are met. Labor force participation is an important way to achieve these goals.

According to activity theory, the active participation of the elderly in social activities can improve life satisfaction and health [7]. When newly retired, older adults suddenly lose most of their social roles, especially at work, and their ties to society diminish, thus leading to the frustration of disengagement. Labor force participation provides a solution to this problem. Through labor force participation, the elderly can strengthen their connection with society, find their role in society, meet their needs of social participation, and significantly improve their health level.

Continuity theory holds that lifestyle in old age is largely influenced by lifestyle in middle age [8,9]. In order to maintain the previous living standard and health status, some elderly people choose to continue to work to maintain the stability of their income sources.

These theories show that labor force participation has a very important impact on the health status of the elderly. From an empirical perspective, it is of great importance to explore this impact.

### 1.1. The Impact of Labor Force Participation on the Health Status of the Elderly

The influencing factors of elderly health have been a long-standing research topic in gerontology, medicine and other disciplines [10,11]. A large number of empirical studies have confirmed that labor participation is one of the important influencing factors of elderly health [12,13,14,15], but these studies have had divergent views on the direction of influence and its transmission channels.

Some studies have suggested that labor force participation improves the health of older people. Using data from the 2015 China Health and Retirement Longitudinal Study (CHARLS), Bai found that the elderly who engaged in labor force participation had mild depressive symptoms [13]. According to Huang and Yu, labor force participation after retirement has a positive impact on the improvement of activities of daily living of the elderly [14]. Wan et al. found that labor force participation plays a positive role in improving the self-reported health, activities of daily living, and chronic diseases of the retired elderly to a certain extent, but they did not find that the depression of the elderly was improved by labor force participation [15].

Retirement is the withdrawal of an older person from the workforce. Many studies have analyzed the impact of retirement on the health status of the elderly. Che and Li conducted a study using data from the China Health and Nutrition Survey (CHNS) and found that retirement had a positive impact on the health status of male employees, which was achieved by strengthening exercise and making lifestyle changes [16]. However, there are many scholars who hold the opposite view. Lee and Kim reported that retirement would harm the health of the elderly [17]. Dong and Zang found that retirement had a negative impact on the health of middle-aged and elderly people by reducing social activities [18]. Feng et al. suggested that retirement led to an increase in BMI in men but had no significant effect on BMI in women because men, especially those in the less educated group, increased their frequency of drinking and reduced physical activity [19]. Dave and Rashad believed that delaying retirement would improve the health status of the elderly and their life happiness [20].

### 1.2. Endogeneity Problems and Solutions

Most scholars believe that health status has a significant positive impact on the labor force participation of the elderly. Van Gameren and Landey conducted empirical analyses using data from Mexico and India, respectively, and found that health status has a strong positive impact on labor force participation of the elderly [21,22]. However, some scholars believe that health status is negatively correlated with labor participation. According to Dwyer and Mitchell, people with poor health status need more medical and health services. In order to meet the increasing demand for medical and health services, they need to participate in work more [23].

The interaction between the labor force participation and physical health of the elderly leads to endogeneity problems to a certain extent. Therefore, scholars have proposed many solutions. Cai and Kalb used simultaneous equations and full information maximum likelihood estimation (FIML) to solve the endogeneity problem between the health status and labor force participation of the elderly, and they compared it with the two-stage least squares method (2SLS). The empirical data showed that the simultaneous equation method was more suitable for studying the relationship between the labor force participation and health status of the elderly [24]. In addition, many scholars have used the same method to deal with the endogeneity problem between the two factors [25,26]. Kalwij and Vermeulen believed that only focusing on one health indicator would greatly deviate estimated results from actual results and that objective health indicators should be comprehensively considered in order to reduce the impact of bias on estimated results [27].

A literature analysis showed that numerous previous studies focused on the impact of elderly health on labor force participation, but few examined the effects of labor force participation on the health status of the elderly and considered the endogeneity issues arising from the mutual causal relationship between the two. This paper fills the corresponding research gap and enriches research on the health of the elderly. Regarding the endogeneity problem, most studies have used the multi-index measuring of the health status of the elderly and simultaneous equations to solve the problem. Different from scholars such as Nwosu and Woolard, who only used self-reported health status as a proxy variable for the health status of the elderly, this paper adopted CES-D 10 and activities of daily living (ADL) to measure the health status of the elderly [28]. In this paper, we address the endogeneity issue based on the work of Wan et al. to investigate the effect of labor force participation on the health status of older adults [15].

## 2. Materials and Methods

### 2.1. Data

Based on the 2018 China Health and Retirement Longitudinal Study (CHARLS), this paper mainly studied the impact of labor force participation on the health status of the elderly aged 60 years and above. The CHARLS was selected in this paper because it provided a variety of proxy variables for the health status of the elderly, which was conducive to improving the credibility of our empirical results. In addition, the sample characteristics provided by CHARLS data are very comprehensive and include individual characteristics, family characteristics, social characteristics, and other contents, which provided sufficient control variables for the empirical study of this paper.

In order to maintain the consistency of the number of sample observations, this paper eliminated samples with missing values and created two sample sets for use in the empirical study of physical health (PH) and mental health (MH). Descriptive statistics showed that these two sample sets were similar to the original sample variable distribution and were representative.

### 2.2. Variable Descriptions

#### 2.2.1. Explained Variable

This paper examined the health status of the elderly (HE) from two dimensions: physical and mental.

1. Physical Health

In this paper, the ADL scale was used to evaluate the physical health status of the elderly [29]. The value of this indicator was determined by the score of the following question: “Do you have difficulty dressing yourself, bathing yourself, eating by yourself, getting up and out of bed by yourself, going to the bathroom by yourself, and controlling your bowel movements because of health and memory reasons?”. If the answer was “no difficulty”, the score was 0; if the answer was “Difficult but still complete”, the score was 1; if the answer was “In trouble and in need of help”, the score was 2; and if the answer was “Unable to complete”, the score was 3. Finally, the scores of the six questions were summed to obtain the total value of physical health. The range of physical health was 0–18. The smaller the total value, the better the physical health status, and the larger the total value, the worse the physical health status.

2. Mental Health

In this paper, the epidemiologic studies depression scale (CES-D 10) proposed by Mohebbi et al. was used to measure the mental health status of the elderly [30]. The value of this indicator was determined by the presence or absence of the following feelings and behaviors in the week prior to the survey: “I had trouble with small things”, “I have a hard time concentrating when I am doing something “, “I feel depressed”, “I feel like it’s a lot of work to do anything“, “I’m full of hope for the future”, “I’m afraid”, “I don’t have a good sleep”, “I am very happy”, “I felt lonely”, and “I think I can continue my life”. If the answer was “little or nothing”, the score was 0; if the answer was “Not much”, the score was 1; if the answer was “Sometimes or half the time”, the score was 2; and if the answer was “Most of the time”, the score was 3 ( “I’m full of hope for the future” and “I am very happy” were scored in opposite ways). The scores of these 10 items were added together to obtain the total score. The value of mental health ranged from 0 to 30. The smaller the value, the better the mental health status, and larger the value, the worse the mental health status.

#### 2.2.2. Explanatory Variable

Referring to the research of Youlu and Ying, this paper chose labor force participation (LFP) as the core explanatory variable [31] and selected three questions as the basis for judging whether an individual had labor force participation behavior: “Have you done farm work or engaged in agricultural activities for your family for at least 10 days in the past year?”, ”In the past year, have you done farm work for another farmer or employer for at least 10 days to earn money?”, and ”Did you work at least an hour last week, excluding anything related to farming?”. If at least one of the answers to the three questions was “yes”, it was regarded as “labor force participation”; otherwise, it was regarded as “no labor force participation”.

#### 2.2.3. Regulated Variable

Referring to the studies of Dong and Zang, Chen and, and Zou et al., taking care of grandchildren and the number of types of social activities of individuals were selected as moderating variables [18,32,33] to explore their role in the impact of labor force participation on elderly health. The question of “In the past year, have you or your spouse spent time caring for your grandchildren and grandchildren?” was used to determine whether they “did not care for their grandchildren” or “did care for their grandchildren”. The number of types of social activities in which an individual participated in the past month was determined based on the question of “Have you engaged in any of the following social activities in the past month?” with the following answer options: “dropping around and socializing with friends”, “playing mahjong, chess, cards and going to the community hall”, “helping for a relative, friend or neighbor who does not live with you“, “dancing, exercising, practicing qigong”, “participating in community activities”, “participating in volunteer activities or charity activities”, “caring for a sick or disabled person who does not live with you“, “going to school or attending training courses, “investing in stocks (funds and other financial securities)”, “surfing the internet”, and “other social activities”.

#### 2.2.4. Control Variables

In this paper, 10 control variables were selected and divided into three groups: individual level, family level, and society level.

1. Personal level

Gender, age, marital status, education and type of residence were selected as control variables at the individual level. When the participants’ marital status was “married, living with spouse” or “married, but because of work and other reasons for not living with a spouse”, respondents were considered married. When participants’ marital status was “separation (not as a spouse living together)”, “divorce”, “bereaved spouse” or “never married”, respondents were considered not married. Education level was a four-category variable, the highest level of education was divided into primary school or below, middle school, high school or technical secondary school, and college or above.

2. Family level

In this paper, the household population was selected as the control variable at the household level. The specific values of family population were obtained according to family information in the data.

3. Social level

In this paper, community security and medical insurance were selected as social control variables. Community security was selected as a continuous variable whose value ranged from 0 to 8 and was defined as the number of home-based and community elderly care services. We determined the number of social security services an individual had received in the past month based on the questions and answers to the following question: “Do you enjoy the following home-based and community elderly care services?” The answer options were “senior service center”, “regular physical examination”, “on-site inspection”, “home hospital beds”, “community care”, “health management”, “recreational activities” and “other”. We used the following question to determine the value of the participants’ health insurance: “Do you personally currently have any of the following medical insurance?”

#### 2.2.5. Instrumental Variable

In this paper, two instrumental variables, both dichotomous, were selected: households enjoying the minimum living guarantee (MLG) and the community average of labor participation (CALP) [11,26].

#### 2.2.6. Descriptive Statistical Analysis of Variables

The descriptive statistical analysis of variables in this paper is shown in Table 1.

### 2.3. Models

#### 2.3.1. Baseline Regression Model

The proxy variables of the health of the elderly (*HE*) selected in this paper were physical health (*PH*) and mental health (*MH*), so a multiple linear regression model was adopted. The specific model was set as follows:

(1) $$HE_{i}=\alpha_{1}+\beta_{1}LFP_{i}+c_{1}X_{i}+\varepsilon_{1}$$

where $HE_{i}$ represents the health of the elderly; $BH_{i}$ and $MH_{i}$ are the physical health and mental health, respectively, of the *i*th individual; $LFP_{i}$ is the labor force participation of the *i*th individual; and $X_{i}$ is the control variable.

#### 2.3.2. Simultaneous Equation Model

According to previous research, labor force participation can impact health in old age and the health of the elderly can affect their labor force participation. Therefore, this paper considered that health in old age and labor force participation are mutually causally related and adopted a simultaneous equation method to solve this problem. The model was set as follows:

(2) $$HE_{i}=\alpha_{21}+\beta_{21}LFP_{i}+c_{21}X_{i}+w_{21}CALP_{i}+\varepsilon_{21}$$

(3) $$LFP_{i}=\alpha_{22}+w_{22}CALP_{i}+c_{22}X_{i}+\varepsilon_{22}$$

(4) $$HE_{i}=\alpha_{23}+w_{23}MLG_{i}+c_{23}X_{i}+\varepsilon_{23}$$

(5) $$LFP_{i}=\alpha_{24}+\beta_{24}HE_{i}+c_{24}X_{i}+w_{24}MLG_{i}$$

In Equation (2), the instrumental variable is the community average of labor participation (CALP), and in Equation (5), the instrumental variable is households enjoying the minimum living guarantee (MLG).

#### 2.3.3. Moderating Effect Model

This paper used taking care of grandchildren (TCG) and social activities (SC) as moderating variables to explore how they affect the relationship between labor force participation (*LFP*) and the health of the elderly (*HE*). The specific model was set as follows:

(6) $$HE_{i}=\alpha_{31}+\beta_{31}LFP_{i}+\beta_{32}LFP_{i}\times TCG_{i}+\beta_{33}TCG_{i}+c_{31}X_{i}+\varepsilon_{31}$$

(7) $$HE_{i}=\alpha_{32}+\beta_{34}LFP_{i}+\beta_{35}LFP_{i}\times SC_{i}+\beta_{36}SC_{i}+c_{32}X_{i}+\varepsilon_{32}$$

where $HE_{i}$ is the health of the *i*th individual, its proxy variables are physical health $PH_{i}$ and mental health $MH_{i}$, TGG_i_ is the *i*th individual caring for grandchildren, and $SC_{i}$ is the participation of the *i*th individual in social activities.

## 3. Results

### 3.1. Health of the Elderly under Different Labor Force Participation Conditions

In this paper, the health status of the elderly with different labor force participation status was calculated for 1535 elderly people with labor force participation behavior and 1715 elderly people without labor force participation behavior in the physical health group and for 2089 elderly people with labor force participation behavior and 2040 elderly people without labor force participation behavior in the mental health group; see Table 2. It can be seen that the number of older people with and without labor force participation behavior was similar. The average physical health status and mental health status of the elderly with labor participation behavior were 0.587 and 9.930, respectively, while the average physical health status and mental health status of the elderly without labor participation behavior were 1.222 and 10.608, respectively. It is known that the elderly with labor participation behavior have better physical and mental health.

### 3.2. Effect of Labor Force Participation on Health in Old Age

In this paper, physical health (PH) and mental health (MH) were used as proxy variables for the health of the elderly to estimate Equation (1) and to obtain the regression results shown in Table 3.

According to the regression results in Table 3, the labor force participation (LFP) of the elderly was negatively correlated with physical health (PH) and mental health (MH), with coefficients of −0.670 and −1.007, respectively. It can be seen that there were significant positive correlations between the labor force participation of the elderly and their physical and mental health status. This paper holds that labor force participation can exercise the body and properly relax mood, thus improving physical and mental health.

Among the individual level control variables, men were found to potentially have better mental health, with a coefficient of −0.483. The mental health of an individual in marriage was found to be worse, with a coefficient of 0.731, because most husband and wife relationships are diluted by time and children affect couples’ feelings. Compared with married older individuals, unmarried individuals showed higher degrees of freedom, without bondage and obsession, so their mood was more relaxed. Individuals with a high education level were found to potentially have better mental and physical health, with coefficients of −0.290, −0.605, −0.981 and −3.273, −4.639, and −5.989. Individuals with a high education level may pay more attention to health maintenance and acquire health knowledge through various channels. Compared with the elderly with a low education level, highly educated individuals may have the habit of reading, which helps them develop a positive attitude towards life and improves their mental health. Individuals living in cities and towns were found to potentially have a worse mental health status than those living in rural areas, with a coefficient of 0.678. 

Among the control variables at the social level, the elderly with more comprehensive community security were found to have better mental health, with a coefficient of −0.778. This is because sound in-home and community-based senior care services provide seniors with a great sense of inner security in case they should become ill. Individuals who have in health insurance were found to have better physical health status and mental health status, with coefficients of −0.513 and −1.278, respectively. This is because when the elderly are sick, health insurance relieves their financial pressure to a certain extent, so they do not have to worry too much about bringing heavy financial pressure to their families due to illness and their psychological burden is smaller. Additionally, mental health status and physical health status affect each other, so having health insurance also improves physical health.

### 3.3. Simultaneous Equation Model

The regression results from Table 3 show that elderly labor force participation, physical health status, and mental health status had significant negative correlations. Because we set labor force participation (LFP) as the explanatory variable and the health of the elderly (HE) was the explained variable, we interpreted our results as revealing that labor force participation improves the physical health and mental health of the elderly. On the one hand, older people in good health are more likely to participate in labor because employers are more likely to choose healthy workers. On the other hand, the health condition of the elderly who participate in labor is better because labor participation fills the inner emptiness of the elderly and enables the elderly to participate in certain sports. Moreover, we found significant positive correlations between physical health and labor participation and between mental health and labor participation. Therefore, the authors of this paper believe that the labor participation and health status of the elderly are mutually causal, and the main explanatory variable is endogenous. However, according to previous research, the health status of the elderly also had an impact on their labor force participation. Therefore, the authors of this paper believe that the health status and labor force participation of the elderly are mutually causal, and this paper adopted a simultaneous equation model to explain this problem. The regression results of simultaneous equations are shown in Table 4.

We examined significant correlations between MLG and health in old age, CALP, and labor force participation, which were not associated with the control variables. MLG was found to be significantly associated with older people’s health because older people from lower income households may not have enough money to treat their illnesses, thus leading to poor physical health. Their mental health was also found to be poor. The community mean of labor participation comes from labor participation, so the community mean of labor participation was shown to be significantly correlated with labor participation. Regarding exogeneity, MLG and the community mean of labor participation were not found to be correlated with the control variables and were entirely derived from outside the model.

MLG was only shown to affect the labor participation of the elderly through their health status. When the elderly are in families with subsistence insurance, poor health status reduces the possibility of labor participation. The community average of labor participation was only shown to affect the health status of the elderly through labor participation. Additionally, these two variables were not found to be related to the error term.

In weak instrumental variable testing, the F value of CALP for LP was 2319.25, that of MLG for BH was 19.99, and that of MLG for MH was 69.75. Therefore, MLG and CALP were not weak instrumental variables.

The simultaneous equation model was used to consider the reciprocal causal relationship between labor force participation (LFP) and health of the elderly (HE), and we separated them to obtain the one-way impact of labor force participation on the health of the elderly and the impact of the health of the elderly on labor force participation.

According to the simultaneous equation regression results shown in Table 4, the coefficient of labor force participation on physical health was −12.581, the coefficient of physical health on labor force participation was −3.120, the coefficient of labor force participation on mental health was −62.809, and the coefficient of mental health on labor force participation was 0.408. It is clear that there was a reciprocal cause–effect relationship between the labor force participation and physical and mental health status of the elderly. This is because labor force participation can improve the health status of older people through appropriate activities and healthy older people actively participate in the labor force.

### 3.4. Analysis of Moderating Effect Results

In this paper, physical health (PH) and mental health (MH) were used as proxy variables for the health of the elderly (HE), and taking care of grandchildren (TCG) and social activities (SC) were used as moderating variables to estimate Equations (6) and (7). To examine whether grandchild care (TCG) and social activities (SC) affected the relationship between the labor force participation (LFP) and health of the elderly (HE), the estimated results are shown in Table 5 and Table 6.

According to Table 5, caring for grandchildren negatively moderated the relationship between labor force participation and physical health among older adults. The labor force participation coefficient was −0.809, and the coefficient of the cross multiplication term was 0.438, which had no significant moderating role in the influence of labor force participation on the mental health of the elderly. It can be seen from Table 6 that participation in social activities had negative moderating effects on the relationship between the labor force participation and physical health status of the elderly and the relationship between the labor force participation and mental health status of the elderly. The labor participation coefficients were −0.858 and −1.410, respectively, and the coefficients of the cross-term were 0.278 and 0.484, respectively. This paper argues that this is because social activities, taking care of grandchildren, and labor force participation can all lead to positive effects on individual health and because there is exclusivity in the time between taking care of grandchildren and labor force participation and between social activities and labor force participation.

### 3.5. Analysis of Heterogeneity

In this section, a heterogeneity analysis is conducted according to the gender and residence area of the interviewed elderly study participants.

Firstly, according to the gender of the elderly, the sample was divided into female elderly and male elderly, and Equation (1) was estimated. The estimation results are shown in Table 7.

As shown in Table 7, labor force participation was found to potentially positively affect the physical health status and mental health status of older individuals in the sample, regardless of gender, but the degree of impact could slightly vary. Overall, the coefficient of labor force participation on physical health status was −0.606 for female older adults with labor force participation behaviors and −0.737 for male older adults with labor force participation behaviors, which shows that labor force participation may have a greater effect on the physical health status of male older adults than female older adults. The coefficient of labor force participation on mental health was −0.671 for female elderly with labor force participation behaviors and −1.362 for male elderly with labor participation behaviors, which shows that labor participation has a greater impact on the mental health of the female elderly than the male elderly.

According to the types of residence area of the elderly, the sample was divided into urban elderly and rural elderly, and Equation (1) was estimated. The estimation results are shown in Table 8.

As shown in Table 8, regardless of the type of residence area, rural or urban, labor force participation was found to potentially have a positive impact on the physical health status and mental health status of older individuals, but the degree of impact could slightly vary. Overall, the coefficient of labor force participation on physical health status was −0.431 for older adults with labor force participation behaviors living in urban areas and −0.724 for older adults with labor force participation behaviors living in rural areas, which shows that labor force participation may have a greater impact on physical health status for older adults living in rural areas than in urban areas. The coefficient of labor force participation on mental health status was −1.042 for older adults with labor force participation behaviors living in urban areas and −1.003 for older adults with labor force participation behaviors living in rural areas, which shows that the degree of influence of labor force participation on the mental health status of older adults in urban and rural areas may not be significantly different.

## 4. Discussion

Based on the 2018 China Health and Retirement Longitudinal Study (CHARLS), this paper selected the ability of daily living (ADL) and the CES-D 10 as the proxy variables for the physical and mental health of the elderly and the labor force participation of the elderly as the explanatory variable of the study. To investigate the effect of labor force participation on the health status of the elderly from multiple dimensions. Firstly, this paper used a multiple linear regression model to conduct benchmark regression to analyze the effect of labor force participation on the health status of the elderly, and it used a simultaneous equation model to explain the reciprocal causal relationship between the health status and labor force participation of the elderly. Then, taking care of grandchildren (TCG) and social activities (SC) were selected as moderating variables to test the moderating effect. The sample was divided into two groups according to the gender and residence area of the elderly in order to explore the heterogeneous role of gender and residence area in the impact of labor force participation on health status.

Through empirical research, this paper found significant positive correlations between labor participation and physical and mental health, which was consistent with the research of Wan et al. [15]. Labor force participation is an important way for the elderly to integrate into society as it can not only allow the elderly to obtain labor remuneration to support their lives and daily medical services but also ensure their normal lives. According to the hierarchy of needs theory, the process of labor force participation also allows the elderly to socialize, learn about emerging things and social development, reduce loneliness, meet social needs, meet respect needs, meet self-actualization needs, and enhance their happiness. At the same time, according to activity theory, labor force participation can strengthen the body and reduce the risk of conditions such as Alzheimer’s disease by enabling the elderly to perform a proper amount of physical and mental work. Therefore, labor force participation can improve the physical and mental health of older people.

It was also found that taking care of grandchildren had a negative moderating effect on the relationship between the labor force participation and physical and mental health of the elderly, and participating in social activities was found to have a negative moderating effect on the relationship between the labor force participation and physical and mental health of the elderly. These conclusions are contrary to the research conclusions of Zhu Y et al. and Wu P [34,35]. Wu P believed that generational care can increase the contact between the elderly and their relatives and friends, as well as increase the possibility of outdoor activities for middle-aged and elderly people, thus improving their physical health. This paper argues that since everyone has a certain amount of time, labor force participation cannot occur at the same time as caring for grandchildren or participating in social activities, and the less time devoted to labor force participation, the less positive effect labor force participation will have on the health status of older adults.

Secondly, labor force participation may have a greater impact on the physical health of elderly men than elderly women and a greater impact on the mental health of elderly women than elderly men. According to continuity theory, the social participation of different elderly individuals is different, so impacts on their health status are also different. Referring to the research results of Wu X et al. [36], from a physiological point of view, men are physically stronger than women and most men undertake most of their family’s manual labor over the course of their lives. Due to the greater exercise compared with women who undertake light manual labor, men’s bodies are stronger and their physical health is better. Women, on the other hand, have a lower family burden and participate in the labor force, which is associated with relaxation and improved mental health.

Finally, labor force participation may have a greater impact on the physical and mental health of the elderly living in rural areas than those living in urban areas. This is because the elderly who live in rural areas for a long time breathe fresh air, take part in farming, and exercise a lot in contrast to those living in cities and towns, where air pollution is a serious issue. Due to convenient transportation and other reasons, the elderly who live in cities and towns have little daily exercise, so the elderly who live in rural areas have better physical health. This is contrary to the research results of Tao H and Zhang X [37], who reported that the elderly living in rural areas had poorer physical health than those living in urban areas due to a lack of health knowledge sources. However, according to the study of Zhang F and Shen C [12], compared with the elderly living in urban areas, the elderly living in rural areas with labor participation are more likely to maintain a positive and optimistic attitude. Now, the urban–rural gap is gradually narrowing, rural areas are also using mobile network, and connections with children and grandchildren are stronger than before, so the loneliness of the elderly has been reduced and their happiness has been improved. Therefore, the impact of labor force participation on the mental health of the urban and rural elderly is not different.

The limitations of this paper are as follows. First of all, this paper measured the health status of the elderly from both physical and psychological aspects instead of explaining the influence of labor participation on the health status of the elderly from a single perspective. However, the ADL scale and CES-D 10 scale were not able to accurately reflect the health status of the elderly. In a study of Chen Y and Sun W, the frailty index was used as a proxy variable for the health status of the elderly [32]. This paper did not consider the comprehensive health status of the elderly, so the frailty index can also be taken into consideration in the future. Secondly, the data used in this paper were cross-sectional data, and the dynamic impact of labor participation on the health status of the elderly was not considered. In the future, multi-period CHARLS data can be used to analyze the dynamic impact between the two factors. Third, although this paper used the simultaneous equation method to solve the endogeneity problem caused by two-way causality, there were missing variables in the selection of control variables. Fourthly, this paper did not discuss the mechanism of labor force participation affecting the health of the elderly. Nevertheless, the results of this study can still provide ideas and reference for people to better understand the relationship between labor force participation and the health of the elderly, as well as its mechanism.

This paper proposes the following directions for future research. First, the weakness index could be added as a proxy variable of elderly health, and the influence of labor force participation on elderly health could be investigated from more dimensions. Second, the sample size could be expanded and multi-period CHARLS data could be used for empirical research to make the research more realistic. Third, as many control variables as possible should be chosen. Fourthly, we could investigate the influence mechanism of labor participation on the health of the elderly.

## 5. Conclusions

The main conclusions of this paper are as follows. (1) This paper found significant positive correlations between the labor participation and physical and mental health of the elderly. According to activity theory, this may have been because participating in labor can properly exercise one’s body and relax one’s mood, thus improving physical and mental health. This holds true even after accounting for the causal effects of labor force participation and the health status of older people. (2) Caring for grandchildren and participating in social activities were found to negatively moderate the relationships between the labor force participation and physical and mental health of older adults because of the time exclusivity between taking care of grandchildren and labor force participation and between social activities and labor force participation. (3) Labor force participation was found to potentially have a greater impact on the physical health of elderly men than elderly women and a greater impact on the mental health of the elderly women than elderly men. This may be because men are physically stronger and women are less stressed at home and can further relax by participating in labor activities. (4) Labor force participation may have a greater impact on the physical health of the elderly in rural areas, possibly because the elderly who live in rural areas for a long time have fresh air and more exercise. A potential reason why the impact of labor force participation on the mental health of the elderly in urban and rural areas showed little difference is because the gap between the elderly in urban and rural areas has narrowed, so the elderly can contact their children at any time and reduce the sense of loneliness.

With the deepening of the aging of Chinese society, relevant policy making also needs to be adjusted. The empirical analysis of the health level of the elderly showed that labor participation can improve the physical and mental health of the elderly. Therefore, China should pay attention to the following issues when formulating future policies.

First, China should accelerate the process of a delayed retirement policy. According to the research results of Yu Xinran and Su Fang [38], some elderly people are still in good health when they retire, which can allow them to continue working. According to activity theory, labor force participation can keep the elderly active, which is not only beneficial to their physical and mental health but could also relieve the government’s pension pressure. Policies could also be introduced to subsidize businesses that hire older people who are in good health and willing to work, as well as to encourage companies to provide employment opportunities for these older people.

Second, we should speed up the process of setting up child-care institutions for children aged 0–3 years to reduce the burden on parents because taking care of grandchildren can occupy the labor participation time of the elderly and labor participation has shown positive effects on their physical and mental health.

Third, policies should reflect gender differences, such as by reducing discrimination against women in the job market. Labor force participation was found to have a positive effect on the physical and mental health of both male and female elderly people. However, gender discrimination still exists in the job market, which is not conducive to the improvement of the health level of the elderly.

## Figures and Tables

**Table 1:** Variables and data statistics.

		Physical Health (PH) (3250)				Mental Health (MH) (4129)			
	Project	Mean	Standard Deviation	Min	Max	Mean	Standard Deviation	Min	Max
Variables									
Health of the elderly		0.922	1.907	0	15	10.265	7.537	0	30
Labor force participation		0.472	0.499	0	1	0.506	0.500	0	1
Taking care of grandchildren		0.362	0.481	0	1	0.381	0.486	0	1
Social activities		0.668	0.902	0	8	0.726	0.969	0	8
Gender		0.494	0.500	0	1	0.492	0.500	0	1
Age		71.243	7.895	60	122	71.274	7.886	60	122
Marital status		0.812	0.391	0	1	0.810	0.392	0	1
Education (junior high school)		0.133	0.339	0	1	0.155	0.362	0	1
Education (high school or junior college)		0.066	0.249	0	1	0.083	0.275	0	1
Education (college degree or above)		0.011	0.106	0	1	0.015	0.120	0	1
Residential area		0.189	0.391	0	1	0.189	0.392	0	1
Number of family members		0.866	1.438	0	11	0.851	1.409	0	11
Community security		0.263	0.581	0	7	0.274	0.598	0	7
Medical insurance		0.960	0.195	0	1	0.966	0.182	0	1

**Table 2:** Health of the elderly under different labor force participation conditions.

	Health Type	Physical Health		Mental Health	
Status of Labor Force Participation		Mean	Number	Mean	Number
With labor force participation		0.587	1535	9.930	2089
Without labor force participation		1.222	1715	10.608	2040

**Table 3:** Health of the elderly under different labor force participation conditions.

Variables	(1)	(2)
	Physical Health	Mental Health
Labor Participation (do not have)	−0.670 *** (0.065)	−1.007 *** (0.230)
Gender (female)	0.003 (0.066)	−0.483 ** (0.231)
Age	−0.006 (0.004)	0.021 (0.015)
Marital status (not married)	0.022 (0.086)	0.731 ** (0.312)
Education (junior high school) (primary and below)	−0.290 *** (0.093)	−3.273 *** (0.283)
Education (high school or junior college) (primary and below)	−0.605 *** (0.100)	−4.639 *** (0.357)
Education (college degree or above) (primary and below)	−0.981 *** (0.128)	−5.989 *** (0.689)
Residence area (rural)	−0.110 (0.076)	0.678 ** (0.294)
Number of family members	0.013 (0.022)	−0.125 (0.079)
Community security (do not have)	−0.047 (0.061)	−0.778 *** (0.181)
Medical insurance (do not have)	−0.513 ** (0.224)	−1.278 * (0.660)
N	3250	4129
R^2^	0.043	0.065

Note: Robust standard errors are in parentheses in the model; ***, ** and * are significant at the 1%, 5% and 10% levels, respectively.

**Table 4:** Simultaneous equation regression results.

Variables	(3)	(4)
	Physical Health	Mental Health
Labor force participation	−12.851 *** (6.425)	−62.809 ** (26.293)
Control variables	Yes	Yes
Community average of labor participation	12.671 *** (6.499)	66.163 *** (26.672)
N	3250	4129
R^2^	0.644	0.007
**Variables**	**Labor Force Participation**	**Labor Force Participation**
Health of the elderly	−3.120 *** (1.251)	0.408 *** (0.077)
Control variables	Yes	Yes
Household enjoying the minimum living guarantee	1.869 *** (0.828)	−1.457 *** (0.314)
N	3250	4129
R^2^	0.879	0.003

Note: Robust standard errors are in parentheses in the model; *** and ** are significant at the 1% and 5% levels, respectively; the control variables in each model have been controlled.

**Table 5:** Regression results of moderating effects (taking care of grandchildren was the moderating variable).

Variables	(5)	(6)
	Physical Health	Mental Health
Labor participation	−0.809 *** (0.087)	−0.954 *** (0.298)
Labor participation × Taking care of grandchildren	0.438 *** (0.123)	0.034 (0.463)
Taking care of grandchildren	−0.532 *** (0.105)	−1.008 *** (0.347)
Control variable	Yes	Yes
N	3250	4129
R²	0.052	0.068

Note: Robust standard errors are in parentheses in the model; *** is significant at the 1% level, respectively; the control variables in each model have been controlled.

**Table 6:** Regression results of moderating effects (the moderating variable was social activities).

Variables	(7)	(8)
	Physical Health	Mental Health
Labor participation	−0.858 *** (0.086)	−1.410 *** (0.290)
Labor participation × Social activities	0.278 *** (0.055)	0.484 ** (0.207)
Social activities	−0.313 *** (0.047)	−1.049 *** (0.151)
Control variable	Yes	Yes
N	3250	4129
R²	0.055	0.076

Note: Robust standard errors are in parentheses in the model; *** and ** and are significant at the 1% and 5% levels, respectively; the control variables in each model have been controlled.

**Table 7:** Differences in the effect of gender on the health status of older adults.

Group	Variables	(9)	(10)
		Physical Health	Mental Health
Female	Labor force participation	−0.606 *** (0.094)	−0.671 ** (0.326)
	Control variable	Yes	Yes
	N	1643	2099
	R²	0.036	0.071
Male	Labor force participation	−0.737 *** (0.092)	−1.362 *** (0.325)
	Control variable	Yes	Yes
	N	1607	2030
	R²	0.054	0.060

Note: Robust standard errors are in parentheses in the model; *** and ** are significant at the 1% and 5% levels, respectively; the control variables in each model have been controlled.

**Table 8:** Differences in the impact of residence type on the health status of the elderly.

Group	Variables	(11)	(12)
		Physical Health	Mental Health
Urban	Labor force participation	−0.431 *** (0.143)	−1.042 * (0.546)
	Control variable	Yes	Yes
	N	613	782
	R²	0.031	0.052
Rural	Labor force participation	−0.724 *** (0.073)	−1.003 *** (0.254)
	Control variable	Yes	Yes
	N	2637	3347
	R²	0.046	0.066

Note: Robust standard errors are in parentheses in the model; *** and * are significant at the 1% and 10% levels, respectively; the control variables in each model have been controlled.

## Data Availability

Data used for this research were provided by the study entitled” China Health and Retirement Longitudinal Study”(CHARLS) managed by the Center for Healthy Aging and Development Studies, Peking University.
